# Genomic distribution and context dependent functionality of novel WRKY transcription factor binding sites

**DOI:** 10.1186/s12864-022-08877-y

**Published:** 2022-09-27

**Authors:** Laureen Christin Arndt, Susanne Heine, Lino Wendt, Emilia Wegele, Jan Titus Schomerus, Jutta Schulze, Reinhard Hehl

**Affiliations:** 1 Institut für Genetik, Technische Universität Braunschweig, Spielmannstr. 7, 38106 Braunschweig, Germany; 2 Institut für Pflanzenbiologie, Technische Universität Braunschweig, Humboldtstr. 1, 38106 Braunschweig, Germany

**Keywords:** Electrophoretic mobility shift assay, Parsley protoplasts, Transcription factors, Transcriptional regulation, Transient gene expression

## Abstract

**Background:**

The WT-boxes NGACTTTN are novel microbe-associated molecular pattern (MAMP)-responsive *cis*-regulatory sequences. Many of them are uncommon WRKY transcription factor (TF) binding sites.

**Results:**

To understand their functional relevance, a genomic distribution analysis of the 16 possible WT-boxes and a functional analysis of a WT-box rich promoter was done. The genomic distribution analysis shows an enrichment of specific WT-boxes within 500 bp upstream of all *Arabidopsis thaliana* genes. Those that harbour a T 5′ to the core sequence GACTTT can also be part of the classic WRKY binding site the W-box TTGACT/C. The MAMP-responsive gene *ATEP3*, a class IV chitinase, harbours seven WT-boxes within its 1000 bp upstream region. In the context of synthetic promoters, the four proximal WT-boxes confer MAMP responsivity while the three WT-boxes further upstream have no effect. Rendering the nucleotides adjacent and in the vicinity of the WT-box core sequence reveals their functional importance for gene expression. A 158 bp long *ATEP3* minimal promoter harbouring the two WT-boxes CGACTTTT, confers WT-box-dependent basal and MAMP-responsive reporter gene expression. The *ATEP3* gene is a proposed target of WRKY50 and WRKY70. WRKY50 negatively regulates MAMP responsivity of the two WT-boxes CGACTTTT, while WRKY70 activates gene expression in a WT-box dependent manner. Both WRKY factors bind directly to the WT-box CGACTTTT.

**Conclusion:**

In summary, WT-boxes are enriched in promoter regions and comprise novel and uncommon WRKY binding sites required for basal and MAMP-induced gene expression. WT-boxes not being part of a W-box may be a missing link for WRKY target gene prediction when these genes do not harbour a W-box.

**Supplementary Information:**

The online version contains supplementary material available at 10.1186/s12864-022-08877-y.

## Background

The W-box TTGACT/C is the classic binding site of the plant specific WRKY TF family [1]. This binding site is enriched in genes involved in systemic acquired resistance and the occurrence of the W-box in promoter regions is a key feature for WRKY target genes [2, 3]. Most of the genomic DNA fragments identified by high throughput TF-binding assays with WRKY factors from *Arabidopsis thaliana*, such as DNA Affinity Purification sequencing (DAP-seq) and chromatin immunoprecipitation contain a W-box in the bound DNA [4, 5]. Therefore, little attention was given so far to deviating WRKY binding sites. Evidence for additional types of WRKY binding sites comes, for example, from chromatin immunoprecipitation sequencing experiments using WRKY18, 33, and 40 in Arabidopsis. For these WRKY factors binding regions were observed that do not harbour W-boxes [5]. Consistent with this, WRKY40 was shown to bind to the sequence TTTTCTA, deviating from the classic W-box [6]. This binding site is similar to the WK-box TTTTCCAC, the binding site of NtWRKY12 in the *PR-1a* promoter from tobacco [7].

To identify novel TF binding sites, experimental approaches may be helpful that identify functional *cis*-regulatory sequences without knowing their interacting TFs. Bioinformatic analyses can propose functional *cis*-sequences in a set of promoter sequences derived from co-regulated genes [8–10]. Co-regulated genes may share common *cis*-regulatory sequences [11]. These common *cis*-sequences can be identified using pattern recognition programs on the promoter region of co-regulated genes and by testing these sequences using reporter gene technology. Subsequently, TFs interacting with these functional *cis*-sequences can be identified. This may lead either to the identification of novel TFs, or to the identification of novel specificities of known TFs such as WRKY factors.

This approach was applied for the identification of novel fungal- and oomycete-responsive sequences in *A. thaliana* [9]. Co-regulated genes upregulated by a diverse set of fungal and oomycete MAMPs were identified using microarray data from the PathoPlant database [12]. Upstream regions of these genes were screened for conserved sequence motifs using BEST, the Binding site Estimation Suit of Tools [13, 14]. To distinguish known *cis*-regulatory sequences from novel ones, the *cis-*regulatory sequence databases AthaMap, PLACE and AGRIS were screened by submitting the *cis*-sequences to the online web tool STAMP [15–18]. As a result, different sequence motifs were identified and were classified into motif families based on their similarities [9]. To investigate the MAMP responsivity of selected *cis*-sequences, tetramers were cloned upstream of the ß-glucuronidase reporter gene in a reporter plasmid and transiently expressed in parsley protoplasts treated with or without the MAMP Pep25 [9, 19, 20]. Pep25, an oligopeptide from a surface glycoprotein of *Phytophthora sojae,* is a standard MAMP used in the parsley protoplast system [21, 22]. This led to the identification of many functional MAMP-responsive *cis*-sequences. One sequence motif is similar to the W-box which is a WRKY TF binding site but harbours a stretch of 3′ T’s [9]. Based on the similarity to W-boxes and the conserved stretch of T’s, these sequences were designated WT-box [23]. The WT-box is an eight bp long sequence and characterized by a conserved GACTTT core sequence while the 5′ and 3′ adjacent nucleotides are variable. Only the WT-box with a T 5′ adjacent to the core sequence GACTTT contains the core sequence TGAC of a classic W-box TTGACT/C [1]. Most of the functional WT-boxes NGACTTTN are not part of the classic W-box. However, many of these WT-boxes were shown to be bound by WRKY TFs. For example WRKY70 binds to the WT-boxes CGACTTTT and AGACTTTT [23–25], WRKY50 binds to GGACTTTT, GGACTTTC, and GGACTTTG [26, 27], and WRKY26 binds to GGACTTTC [28]. Therefore, these *cis*-sequences increase the repertoire of known WRKY binding sites and may be useful to predict WRKY target genes.

To gain more insight into the genomic distribution and possible enrichment of certain WT-boxes in promoter regions, the distribution of all sixteen possible WT-boxes NGACTTTN in *A. thaliana* was investigated. This revealed the enrichment of specific WT-boxes within a 500 bp upstream region of all nuclear genes. To investigate the functional importance of specific WT-boxes for MAMP responsivity, seven WT-boxes from a WT-box rich promoter of the MAMP-responsive class IV chitinase *ATEP3* (At3g54420) were investigated experimentally. This analysis uncovered functionally relevant nucleotides within the WT-box and identified sequences adjacent to the WT-box which influence functionality. Based on the prediction of *ATEP3* as a target of WRKY50 and WRKY70, both TFs are shown to interact with the WT-boxes CGACTTTT, essential for MAMP-responsive and basal gene expression in the *ATEP3* minimal promoter. Using chromatin immunoprecipitation sequencing data W-box free target genes of WRKY18, 33, and 40 are shown to harbour WT-boxes in their promoters.

## Results

### Genomic distribution of WT-boxes in *A. thaliana*

WT-boxes were identified as novel *cis*-regulatory sequences for MAMP-responsive gene expression. To play a role in gene expression regulation, functional *cis*-regulatory sequences are expected to occur within the promoter region of the genes. Although the length of Arabidopsis promoters may vary, most of the regulatory sequences occur within a region of 500 bp upstream to the transcription start site [29, 30]. To analyse the genomic distribution of all possible WT-boxes NGACTTTN, the number and frequency of WT-boxes were determined in the whole *Arabidopsis thaliana* genome and in the region 500 bp upstream of the transcription start site (TSS). Furthermore, the frequency of each WT-box was compared with the expected frequency. Table 1 shows the absolute number of each WT-box either genome wide or within the 500 bp upstream region of all nuclear genes. Furthermore, the observed and expected frequencies are shown. In cases where the WT-box core sequence GACTTT harbours a 3′ adjacent T and either a 5′ adjacent A, C, or T, these sequences occur more often within the 500 bp upstream region and genome-wide than in the other cases. The highest numbers of WT-boxes are 2319 in the 500 bp region and 12,436 genome-wide for the WT-box TGACTTTT. This WT-box harbours the core sequence TGAC for the W-box and indicates its functional conservation within possible WRKY binding sites.

**Table 1:** Number and frequency of WT-boxes in the *A. thaliana* genome and in a region 500 bp upstream of the genes

WT-Box	Observed numbers within 500 bp upstream	Observed numbers Genome wide	Frequency 500 bp upstream of all genes	Frequency genome wide	Expected frequency
AGACTTTA	969	7633	58158E-05	56261E-05	388968E-05
AGACTTTC	563	5432	337905E-05	40038E-05	188307E-05
AGACTTTG	893	8106	535966E-05	59748E-05	188307E-05
AGACTTTT	1697	10,639	0,000101852	78418E-05	388968E-05
CGACTTTA	327	2525	196261E-05	18611E-05	188307E-05
CGACTTTC	239	2121	143444E-05	15633E-05	911633E-06
CGACTTTG	379	3259	227471E-05	24021E-05	911633E-06
CGACTTTT	652	3683	391321E-05	27147E-05	188307E-05
GGACTTTA	348	2790	208865E-05	20565E-05	188307E-05
GGACTTTC	255	2938	153047E-05	21655E-05	911633E-06
GGACTTTG	428	4440	25688E-05	32726E-05	911633E-06
GGACTTTT	660	4387	396123E-05	32336E-05	188307E-05
TGACTTTA	1069	7216	641599E-05	53188E-05	388968E-05
TGACTTTC	717	5519	430333E-05	4068E-05	188307E-05
TGACTTTG	1155	9374	693215E-05	69094E-05	188307E-05
TGACTTTT	2319	12,436	0,000139183	91663E-05	388968E-05

**Fig. 1:**
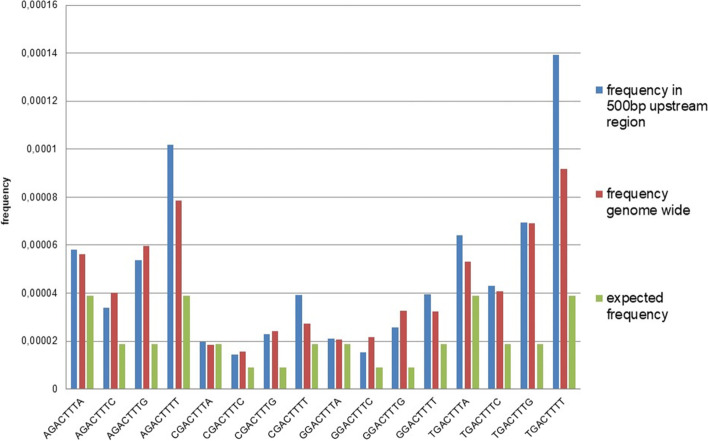
Observed frequencies of the 16 WT-boxes within 500 bp upstream of all nuclear genes and genome wide in *A. thaliana* and expected frequencies. A binomial distribution analysis shows that the increased frequency of all sequences, except CGACTTTA and GGACTTTA, in the upstream region is highly significant when compared to the expected frequency (*p* < 0,01). When the frequency in the upstream region is compared to the frequency genome wide, there is a significant increase for the sequences AGACTTTT, CGACTTTT, GGACTTTT, TGACTTTA, and TGACTTTT (*p* < 0,05)

To determine if certain WT-boxes are significantly enriched, the frequency of each WT-box was determined and compared with the frequency expected if the WT-boxes were occurring randomly. The calculation of the expected frequencies takes the GC/AT content into account (Methods). Table 1 shows the observed frequencies for each WT-box in the genome, in the 500 bp upstream region, and their expected frequencies. Figure 1 shows a graphic comparison of these frequencies for all 16 WT-boxes. A binomial distribution analysis shows a highly significant increase in the frequency of all sequences, except CGACTTTA and GGACTTTA, in the upstream region compared to the expected frequencies (*p* < 0,01). Furthermore, when the frequency in the upstream region is compared to the frequency genome wide, there is a significant enrichment of the sequences AGACTTTT, CGACTTTT, GGACTTTT, TGACTTTA, and TGACTTTT (*p* < 0,05). Taken together, WT-boxes harbouring a T 3′ and/or 5′ to the GACTTT core sequence occur more often compared to the other WT-boxes and most of the WT-boxes, except two, are significantly enriched in the 500 bp upstream region of the genes.

### Four of the seven WT-boxes from the *ATEP3* upstream region confer MAMP responsivity in the context of synthetic promoters

**Fig. 2:**
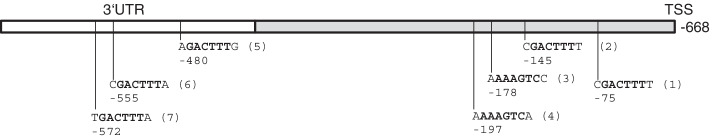
Schematic representation of a 668 bp *ATEP3* upstream region with the positions of the identified WT-boxes. Positions of the seven WT-boxes (1 through 7) upstream of the transcription start site (TSS) are indicated. Three WT-boxes (5 through 7) are located within the 3′ untranslated region (3’UTR) of the upstream gene

**Fig. 3:**
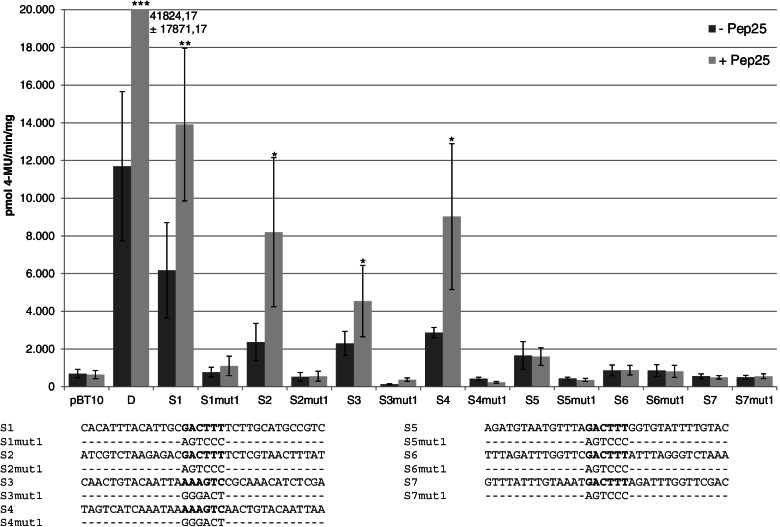
The four proximal WT-box containing sequences from the *ATEP3* upstream region confer WT-box-dependent reporter gene expression. Transient reporter gene assays in parsley protoplasts after transformation and treatment with and without Pep25 (light grey bar and dark grey bar, respectively). The reporter plasmids used for transformation harbour four copies of the indicated sequences upstream of the *uid*A (GUS) reporter gene. Relative GUS expression (pmol 4-MU/min/mg protein) and standard deviations were determined from eleven (pBT10, D) or three (S1, S1mut1, S2, S2mut1, S3, S3mut, S4, S4mut1, S5, S5mut1, S6, S6mut1, S7, S7mut1) independent experiments with technical duplicates, respectively. Statistical differences between experiments were determined with a t-test in microsoft excel. Statistically significant Pep25 inductions are indicated with one (*p* < 0.05), two (*p* < 0.01), and three (*p* < 0.001) asterisks. Sequences of the seven monomers (S1 through S7) and the seven sequences in which the WT-box are mutated are shown. The WT-box core sequence GACTTT is marked in bold. Altered nucleotides in the mutations are shown and unaltered nucleotides are not shown (−)

To investigate the functional relevance of individual WT-boxes, preferably without being part of a W-box, a MAMP-responsive gene harbouring a large number of WT-boxes in its upstream region was chosen. To identify such a gene, the web tool Patmatch at The Arabidopsis Information Resource (TAIR) was used to search for the WT-box core sequence GACTTT within 1000 bp upstream of all annotated genes (Methods). Two genes have seven WT-box core sequences in this region, which is the maximum number detected. *ATEP3* (At3g54420) was chosen for further analysis. *ATEP3* is a class IV chitinase and is upregulated by a large number of MAMPs [12]. According to TAIR the gene “is expressed during somatic embryogenesis in ‘nursing’ cells surrounding the embryos but not in embryos themselves. The gene is also expressed in mature pollen and growing pollen tubes until they enter the receptive synergid, but not in endosperm and integuments as in carrot. Post-embryonically, expression is found in hydathodes, stipules, root epidermis and emerging root hairs” [31–33]. Figure 2 shows a schematic representation of the upstream region of *ATEP3* with all seven WT-boxes. Four of them occur within the 200 bp upstream region of the gene, while the other three are located further distal within the 3′ untranslated region (3’UTR) of the upstream gene. Two WT-boxes (Positions − 178 and − 197) are in the opposite orientation than the other five. Figure 3 shows the nucleotides surrounding each WT-box. Only one WT-box (S4) is part of a classic W-box TTGACT.

To test the functionality of all seven WT-boxes, 34 bp long *cis*-sequences corresponding to the respective wild type sequence and harbouring the WT-box in central position were synthesized. Also, all sequences harbouring a mutation of the WT-box core sequence were synthesized. These 14 sequences are designated S1 through S7 and S1mut1 through S7mut1 (Fig. 3). The most proximal WT-box containing sequence is designated S1 while the most distal sequence is designated S7 (Fig. 2). The 14 sequences were cloned in their native orientation as tetramers upstream to the minimal promoter of the GUS gene in pBT10GUS-d35SLUC [9]. Transient reporter gene experiments were carried out as described, using the parsley protoplast system and the MAMP Pep25 [20]. As a positive control, pBT10GUS-d35SLUC with four copies of the D-element, shown to confer strong Pep25-responsive reporter gene expression, was used [34]. The empty vector was used as a negative control. Figure 3 shows the results of the quantitative GUS assays. The sequences S1 through S4 show Pep25-responsive gene expression. Pep25 responsivity is abolished with a mutation of the WT-box in all four sequences (S1mut1 through S4mut1). In contrast, the three WT-boxes located further upstream (S5 through S7) and their mutations (S5mut1 through S7mut1) do not confer Pep25-responsive reporter gene expression. Therefore, the four WT-boxes located proximal to the gene in the 200 bp *ATEP3* upstream region, are required for reporter gene expression. These WT-boxes act in a distance independent manner because all synthetic promoters harbour the WT-boxes in the reporter plasmid in the same distance to the reporter gene. Consequently, the sequence of the WT-boxes or their sequence context may be the main feature distinguishing the four functional ones from the three non-functional WT-boxes. In summary, the WT-boxes CGACTTTT (S1, S2), GGACTTTT (S3), and TGACTTTT (S4) are required for MAMP responsivity in the context of synthetic promoters.

### The 5′ and 3′ adjacent nucleotides to the WT-box core sequence GACTTT contribute to their functionality

When the four MAMP-responsive WT-boxes of the *ATEP3* gene are compared with the three non-responsive ones, the four functional WT-boxes located within the 200 bp upstream region all feature a T 3′ to the core sequence GACTTT. The three non-functional WT-boxes further upstream do not harbour a T 3′ to the WT-box core sequence (Figs. 2 and 3).

**Fig. 4:**
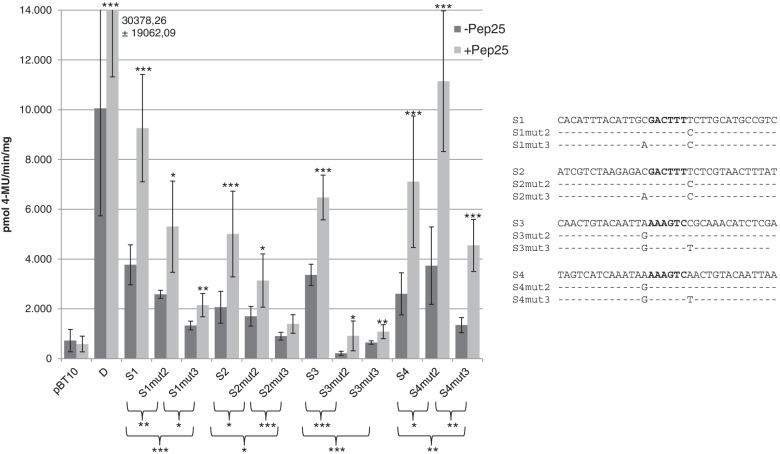
WT-box core sequence adjacent nucleotides contribute to MAMP-responsive gene expression of the four proximal WT-boxes from the *ATEP3* upstream region. Transient reporter gene assays in parsley protoplasts were done as described in Fig. 3. Relative GUS expression and standard deviations were determined from twelve (pBT10, D), nine (S4), seven (S1, S2), four (S2mut3, S3, S3mut2, S3mut3), or three (S1mut2, S1mut3, S2mut2, S4mut2, S4mut3) independent experiments with technical duplicates, respectively. Statistical differences between experiments were determined with a t-test in microsoft excel. Statistically significant Pep25 inductions are indicated with asterisks as described in Fig. 3. Sequences of the four monomers (S1 through S4) and the eight sequences in which the WT-box core sequence adjacent nucleotides are mutated are shown. The WT-box core sequence is marked in bold. Altered nucleotides in the mutations are shown and unaltered nucleotides are not shown (−)

The first question was, whether a mutation of the T 3′ adjacent to the WT-box core sequence has an effect on the functionality of the four proximal WT-boxes. In these four cases, the T 3′ adjacent to the core sequence GACTTT was changed to a C. These mutations are designated S1mut2 through S4mut2 and tetramers of these sequences were tested in the parsley protoplast system as mentioned before. As shown in Fig. 4 all sequences harbouring the 3′ adjacent C instead of the T maintain their MAMP responsivity but basal and MAMP-responsive expression is significantly lower than in the unmutated sequences, except for S4mut2. The lack of a negative effect in S4mut2 may be explained by the presence of a perfect W-box TTGACT which is unaffected by the mutation. In summary, the 3′ T has a quantitative effect on gene expression in the WT-boxes CGACTTTT (S1, S2) and GGACTTTT (S3).

To further investigate the effect of the 5′ adjacent nucleotide in these four WT-boxes, a second mutation was introduced in addition to the mutated 3′ T. For this, the C 5′ adjacent to the core sequence (S1mut2, S2mut2), the G 5′ adjacent to the core sequence (S3mut2) and the T 5′ adjacent to the core sequence (S4mut2) were changed into an A yielding mutations S1mut3 through S4mut3 (Fig. 4). Tetramers of these sequences were tested in the parsley protoplast system as well. As shown in Fig. 4, S1mut3 through S3mut3 further reduced the MAMP responsivity of the WT-boxes CGACTTTT (S1, S2) and GGACTTTT (S3) significantly. S4mut3 still shows significant MAMP responsivity, lower than S4mut1 and S4mut2. Therefore, sequence AGACTTTC in S4mut3 is sufficient for MAMP responsivity. In summary, the mutation analysis of the three WT-boxes CGACTTTT (S1, −S2) and GGACTTTT (S3) shows the requirement of the 5′ and 3′ nucleotides adjacent to the WT-box core sequence GACTTT for their functionality.

### Rendering non-functional WT-boxes into functional ones

**Fig. 5:**
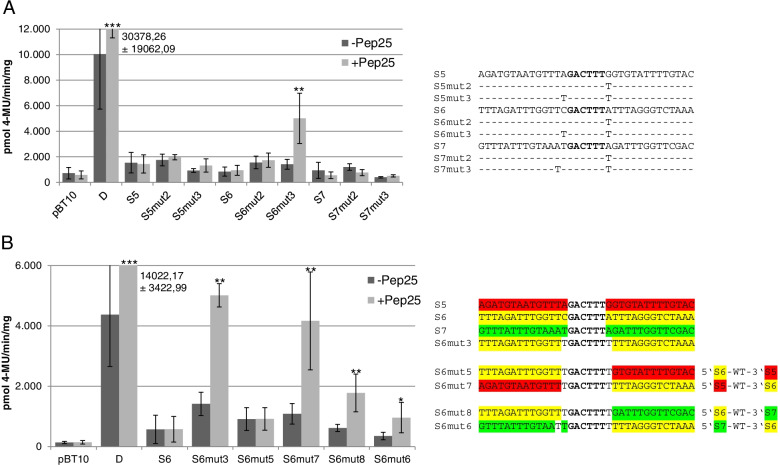
Changing non-functional WT-boxes into functional ones. Transient reporter gene assays in parsley protoplasts were done as described in Fig. 3. Relative GUS expression and standard deviations were determined from twelve (pBT10, D), nine (S5, S6, S7), three (S5mut2, S5mut3, S6mut2, S6mut3, S7mut2, S7mut3) independent experiments (A) and from four (pBT10, D, S6, S6mut5, S6mut6, S6mut8) or three (S6mut3, S6mut7) independent experiments (B) all with technical duplicates. Statistical differences between experiments were determined with a t-test in microsoft excel. Statistically significant Pep25 inductions are indicated with asterisks as described in Fig. 3. **A** Changing the WT-boxes in S5, S6, and S7 into a WT-box harbouring W-box TTGACTTTT only renders S6 MAMP-responsive (S6mut3). Sequences of the three monomers (S5, S6, and S7) and the six sequences in which the WT-box core sequence adjacent nucleotides were mutated are shown. **B** Exchanging the sequences 5′ and 3′ to the WT-box TTGACTTTT in S6mut3 with the respective sequences from S5 and S7 affect MAMP responsivity. The sequences S5 through S7 are shown and 5′ and 3′ regions in each sequence are indicated in red (S5), yellow (S6), and green (S7). The colour code was maintained to indicate exchanged regions in the four altered sequences

As mentioned before, a common difference between the three non-functional WT-boxes within the 3′ UTR of the upstream gene and the four functional ones further proximal to the TSS of the *ATEP3* gene is the lack of a T 3′ adjacent to the WT-box core sequence GACTTT (Figs. 2 and 3). Therefore, it was analysed if changing the 3′ adjacent nucleotides into a T is sufficient to render the non-functional WT-boxes functional. These mutations, S5mut2 through S7mut2, were analysed for MAMP responsivity in the parsley protoplast system as shown in Fig. 5A. These changes did not have an effect on the functionality of these three WT-boxes. Neither the unchanged sequences S5 through S7 nor the sequences S5mut2 through S7mut2 show MAMP responsivity (Fig. 5A). Similar to the analysis of the functional WT-boxes proximal to the TSS of the *ATEP3* gene, also the 5′ adjacent nucleotides of the WT-box core sequence in S5mut2 and S6 mut2 were changed into a T. In case of S7mut2, the A 5′ adjacent to the WT-box TGACTTTT was changed into a T. These mutants were designated S5mut3, S6mut3, and S7mut3 (Fig. 5A) These mutations were expected to yield MAMP-responsive WT-boxes, because all three mutated WT-boxes display a perfect W-box TTGACT as part of the WT-boxes TGACTTTT (Fig. 5A). Surprisingly, only one of the three WT-boxes was rendered MAMP-responsive (S6mut3) while the other two, S5mut3 and S7mut3, although harbouring the same WT-box containing W-box TTGACTTTT, do not show MAMP-responsive gene expression (Fig. 5A). Therefore, sequences adjacent to the WT-boxes in S5mut3, S6mut3 and S7mut3 may have a regulatory effect on their MAMP responsivity.

To further investigate the role of the TTGACTTTT adjacent sequences in these synthetic promoters, the sequences 5′ and 3′ to the sequence TTGACTTTT in S6mut3 were exchanged with the respective 5′ and 3′ sequences of S5mut3 and S7mut3. To better illustrate these exchanges, the 5′ and 3′ sequences of S6mut3 are shown in yellow, the ones from S5mut3 are shown in red and the ones from S7 are shown in green (Fig. 5B). Interestingly, when the 3′ sequence from S6mut3 was exchanged against the 3′ sequence from S5, MAMP responsivity is completely abolished (S6mut5, Fig. 5B). This either shows a requirement of the 3′ sequence from S6mut3 for its MAMP responsivity or a repressive effect of the 3′ sequence from S5mut3. The requirement of the 3′ sequence from S6mut3 for its MAMP responsivity is supported by the exchange of the 5′ sequence of S6mut3 against the 5′ sequence from S5mut3. In this case the exchange has no significant effect on its MAMP responsivity (S6mut7, Fig. 5B). This supports the requirement of the sequence 3′ to the sequence TTGACTTTT in S6mut3 for its MAMP responsivity. However, a different result is obtained when the 3′ sequence from S6mut3 was exchanged against the 3′ sequence from S7. In this case MAMP responsivity is not completely abolished (S6mut8, Fig. 5B). A similar result was obtained when the 5′ sequence from S6mut3 was exchanged against the 5′ sequence from S7. In this case MAMP responsivity is also not completely abolished (S6mut6, Fig. 5B). This indicates a positive effect of the 5′ and 3′ sequences of S6mut3 when they are exchanged against the 5′ and 3′ sequences in S7mut3 (S6mut6 and S6mut8, Fig. 5B). In summary, these experiments show the importance of the sequence context of the WT-box TTGACTTTT in S6mut3 for its MAMP responsivity.

### A 158 bp promoter fragment of *ATEP3* is sufficient for basal and MAMP-responsive gene expression harbouring two WT-boxes CGACTTTT essential for gene expression

**Fig. 6:**
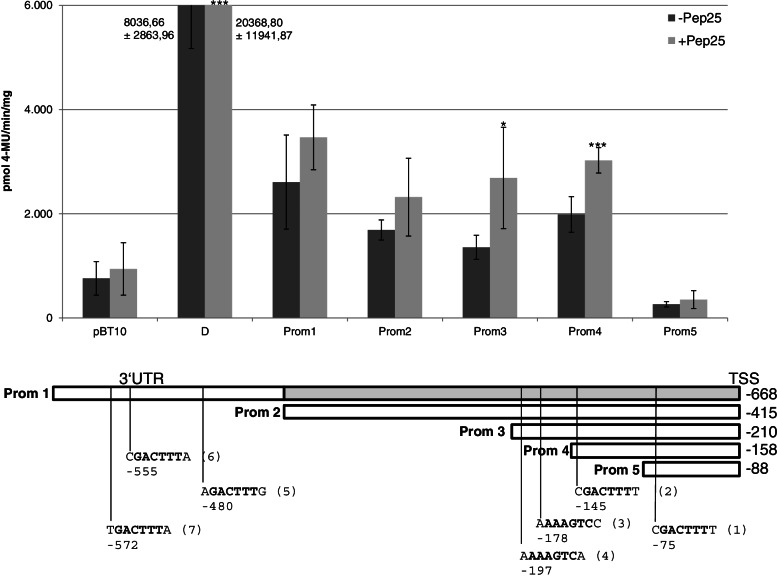
A 158 bp minimal promoter of the *ATEP3* upstream region confers MAMP responsivity. Five promoter fragments with the position of the seven WT-boxes are shown. All promoter fragments were cloned upstream of the *uidA* reporter gene and transient reporter gene experiments were done as described in Fig. 3. Relative GUS expression and standard deviations were determined from three independent experiments with technical duplicates. Statistical differences between experiments were determined with a t-test in microsoft excel. Statistically significant Pep25 inductions are indicated with asterisks as in Fig. 3

The previous results were obtained in the context of synthetic promoters in which WT-box containing sequences were analysed as tetramers. To determine the functionality of the WT-boxes in the context of the native promoter, five promoter fragments were linked to the ß-glucuronidase (GUS) gene in the plasmid pBT10GUS-d35SLUC after removing the TATA-box in the minimal promoter upstream to the GUS gene (Methods). This aims to determine the *ATEP3* minimal promoter required for basal and MAMP-responsive gene expression. Transient reporter gene expression experiments were carried out as described above. Figure 6 shows promoter fragments in size from 668 to 158 bp to confer basal and MAMP-responsive gene expression, while an 88 bp long promoter fragment neither shows basal nor MAMP-induced reporter gene expression above the negative control pBT10. Therefore, 158 bp of the promoter, harbouring the two WT-boxes CGACTTTT are sufficient for MAMP responsivity and basal gene expression (Prom4, Fig. 6).

**Fig. 7:**
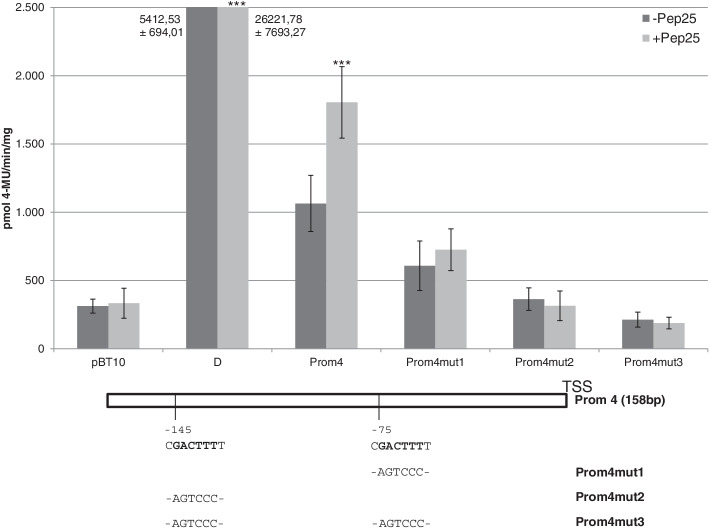
The WT-boxes in the 158 bp long *ATEP3* minimal promoter are essential for basal and MAMP-responsive gene expression. Promoter fragment Prom4 and three mutations in which a single or both WT-boxes were mutated are shown schematically. These were cloned upstream of the *uidA* reporter gene and transient reporter gene experiments were done as described in Fig. 3. Relative GUS expression and standard deviations were determined from three independent experiments with technical duplicates. Statistical differences between experiments were determined with a t-test in microsoft excel. Statistically significant Pep25 inductions are indicated with asterisks as in Fig. 3

To investigate the role of the two WT-boxes CGACTTTT in this region for basal and MAMP-responsive gene expression, single and double mutations were introduced into the WT-boxes and the effect of these mutations on reporter gene activation were analysed using the parsley protoplast system. Figure 7 shows the result of these analyses. Compared to the wild type 158 bp promoter fragment (Prom4), single mutations of the WT-boxes (Prom4mut1 and mut2) abolish MAMP-responsive gene expression while a promoter fragment carrying mutations in both WT-boxes (Prom4mut3), no longer allows background reporter gene expression (Fig. 7). Both WT-boxes CGACTTTT in the 158 bp minimal promoter region are essential for basal and MAMP-responsive gene expression.

### WRKY50 and WRKY70 are proposed regulators of *ATEP3*

The WT-box CGACTTTT, essential for MAMP-responsive gene expression of the *ATEP3* minimal promoter, was previously shown to interact with WRKY70, and WRKY50 is also a factor which binds to WT-boxes [23, 27]. To investigate if the *ATEP3* gene is a proposed target of both WRKY factors, the Cistrome database was consulted [4]. This database contains proposed target genes of Arabidopsis TFs identified by DNA Affinity Purification sequencing (DAP-seq). Genomic fragments bound by the employed TFs were sequenced and constitute the basis for target gene prediction. The proposed target gene sets of WRKY50 and WRKY70 can be downloaded from the Cistrome database. Both sets of target genes contain the *ATEP3* chitinase [4]. The DAP-seq based identification of *ATEP3* as a target gene for WRKY50 and WRKY70 does not mean the two essential WT-boxes in the 158 bp minimal promoter are located in the fragments bound by WRKY50 and WRKY70. To investigate this, the genome browser at the Cistrome database was used to determine if these WT-boxes occur in the fragments identified by DAP-seq. This analysis confirmed both WT-boxes to be present within WRKY50- and WRKY70-bound DNA fragments [4]. Therefore, it was investigated if both WRKY factors can interact with the WT-boxes CGACTTTT and if they activate or repress reporter gene expression by interacting with the WT-boxes of the minimal promoter.

### WRKY50 represses MAMP-responsive reporter gene activation and directly binds to the two WT-boxes from the *ATEP3* minimal promoter

**Fig. 8:**
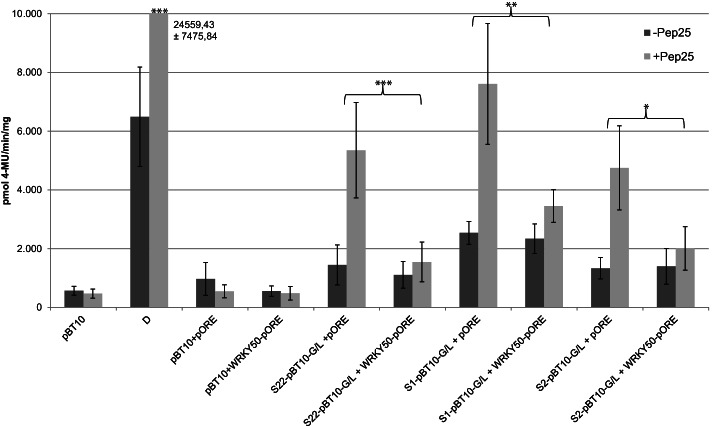
WRKY50 negatively regulates MAMP-responsive reporter gene expression through WT-box containing synthetic promoters. Transient reporter gene assays in parsley protoplasts after co-transformation of the effector plasmid WRKY50-pORE or the empty vector (pORE) with reporter plasmids harbouring four copies of the indicated sequences upstream of the *uid*A (GUS) reporter gene. Relative GUS expression and standard deviations were determined from six (pBT10, D, pBT10 + pORE, pBT10 + WRKY50pORE), twelve (S22 + pORE, S22 + WRKY50pORE), or three (S1 + pORE, S1 + WRKY50pORE, S2 + pORE, S2 + WRKY50pORE) independent experiments with technical duplicates, respectively. Statistical differences between experiments are indicated with one (*p* < 0.05), two (*p* < 0.01), and three (*p* < 0.001) asterisks. The sequences of S1 and S2 are shown in Fig. 3

To analyse the interaction of WRKY50 and WRKY70 with the WT-boxes in the *ATEP3* minimal promoter, transient reporter gene expression assays and gel-shift experiments were performed. Co-transformation experiments were done with a WRKY50- or WRKY70-expressing effector plasmid [23, 27] and with reporter plasmids harbouring four copies of the WT-boxes S1 and S2 previously employed for investigating MAMP-responsive gene expression (Fig. 3). As a control, a co-transformation with the empty effector plasmid (pORE) and the reporter plasmid void of *cis*-regulatory sequences (pBT10) was performed as well. Furthermore, the experiments were done in the presence and absence of the MAMP Pep25. As an additional control, sequence S22 previously shown to be negatively regulated by WRKY50 was also tested [27]. Figure 8 shows the result of these transient expression experiments for WRKY50. The co-transformation of WRKY50 with the three synthetic promoter constructs S22, S1, and S2 did not show an upregulation of reporter gene expression in the presence of WRKY50 (compare S22, S1, and S2 + pORE without Pep25 with S22, S1, and S2 + WRKY50-pORE without Pep25, Fig. 8). In contrast, co-transformation of WRKY50 has a negative effect on Pep25-induced reporter gene expression. Figure 8 shows a negative effect of WRKY50 on MAMP-induced reporter gene activity when the synthetic promoter constructs harbour tetramers of S1 and S2 and the control sequence S22 from the previous study (compare S22, S1, and S2 + pORE with Pep25 with S22, S1, and S2 + WRKY50-pORE with Pep25). These results indicate a negative effect of WRKY50 on MAMP-responsive reporter gene expression, probably by binding to the WT-boxes CGACTTTT in S1 and S2.

**Fig. 9:**
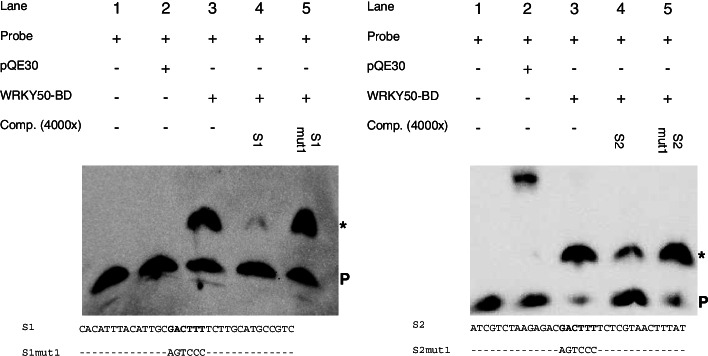
Binding of WRKY50BD to the sequences S1 and S2 requires the WT-boxes CGACTTTT. Lanes 1: free probe. Lanes 2: free probe plus purified protein extract of *E. coli* not expressing WRKY50BD. Lanes 3: free probe plus purified WRKY50BD. Lanes 4: free probe, purified WRKY50BD, and unlabelled competitor (S1 or S2, respectively) in a 4000-fold molar excess. Lanes 5: free probe, purified WRKY50BD, and unlabelled mutations S1mut1 or S2mut1, respectively in a 4000-fold molar excess. The sequences of S1, S1mut1, S2, and S2mut1 are shown. Unchanged nucleotides in S1mut1 and S2mut1 are indicated by a dash (−). A P designates the position of the free probe and a specific DNA-protein complex is marked with an asterisk (*). The gel shifts were done three times and one example is shown. The original unprocessed figures are shown in Fig. S1 and Fig. S2

To show binding of WRKY50 to the WT-boxes CGACTTTT in S1 and S2, electrophoretic mobility shift assays (EMSA) were performed. For this, a truncated version of WRKY50 harbouring the DNA binding domain (WRK50BD) was expressed in *E. coli* and purified (Methods). In an earlier report, the full length WRKY50 could not be used for in vitro binding studies but the 88 bp C-terminal DNA binding domain (WRKY50BD) produced a shift with an 80 bp *PR1* promoter fragment [26]. Therefore, the EMSA analysis was performed with WT-box containing sequences S1 and S2 and purified WRKY50BD. Figure 9 shows a shifted complex observed in the presence of WRKY50BD in the case of both sequences S1 and S2 (lanes 3). This signal can be abolished or strongly reduced with an excess of unlabelled S1 and S2 (Fig. 9, lanes 4). However, the sequences in which the WT-box is mutated (S1mut1 and S2mut1) are no longer able to abolish the shift (Fig. 9, lanes 5). In summary, WRKY 50 negatively regulates the MAMP-responsivity of the synthetic promoters harbouring the WT-boxes CGACTTTT by directly binding to these WT-boxes.

### WRKY70 activates reporter gene expression by interacting with the WT-boxes of the *ATEP3* minimal promoter

**Fig. 10:**
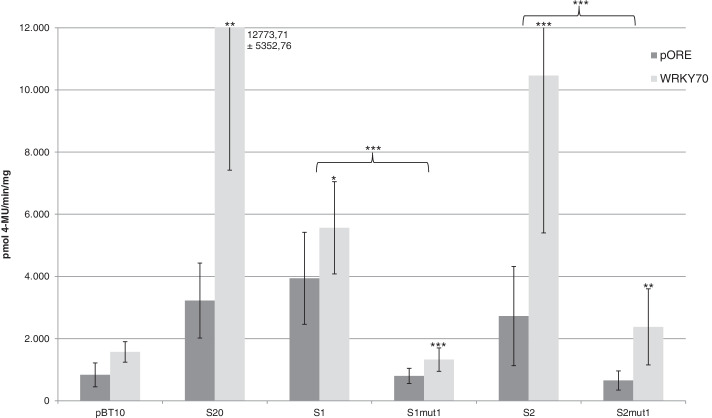
WRKY70 activates reporter gene expression through WT-box containing synthetic promoters S1 and S2. Transient reporter gene assays in parsley protoplasts after co-transformation of the effector plasmid WRKY70-pORE or the empty vector (pORE) with reporter plasmids harbouring four copies of the indicated sequences upstream of the *uid*A (GUS) reporter gene. Relative GUS expression and standard deviations were determined from three (pBT10, S20, S1, S1mut1, S2, S2mut1) independent experiments with technical duplicates, respectively. Statistical differences between experiments are indicated with one (*p* < 0.05), two (*p* < 0.01), and three (*p* < 0.001) asterisks. The sequences of S1, S1mut1, S2, and S2mut1 are shown in Fig. 3

To analyse the effect of WRKY70 on reporter gene expression, co-transformation experiments were done with a WRKY70-expressing effector plasmid [23] and with reporter plasmids harbouring four copies of the WT-boxes S1 and S2. As a control, a co-transformation with the empty effector plasmid (pORE) and a reporter plasmid void of *cis*-regulatory sequences (pBT10) was done as well [23, 27]. As an additional control, sequence S20, harbouring the same WT-box CGACTTTT was employed because four copies of this sequence showed upregulated gene expression in the presence of WRKY70 [23]. To investigate if the WT-boxes in S1 and S2 are required for gene expression, the same experiment was done in which the WT-boxes were mutated (S1mut1 and S2mut1). Figure 10 shows the result of the co-transformation of WRKY70 with the synthetic promoter constructs S20, S1, and S2, revealing a highly significant upregulation of reporter gene expression with S20 and S2 but a low upregulation with S1. This is surprising, since all three sequences harbour the same WT-box CGACTTTT. When this WT-box is mutated in S1 and S2, reporter gene expression is significantly lower. Therefore, both WT-boxes are required for WRKY70-activated reporter gene expression.

**Fig. 11:**
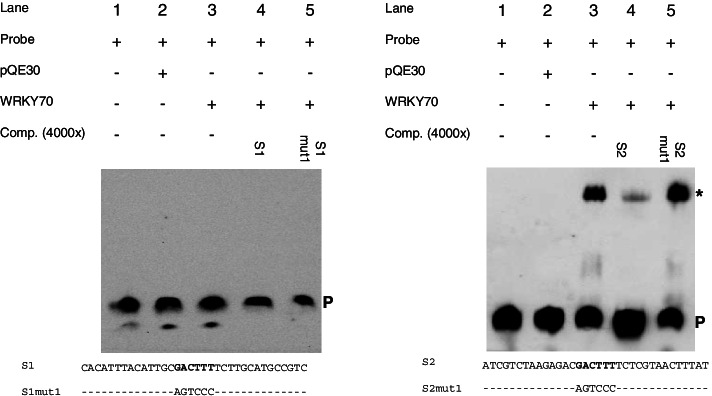
Binding of WRKY70 to the sequence S2 requires the WT-boxes CGACTTTT. Lanes 1: free probe. Lanes 2: free probe plus purified protein extract of *E. coli* not expressing WRKY70. Lanes 3: free probe plus purified WRKY70. Lanes 4: free probe, purified WRKY70, and unlabelled competitor (S1 or S2, respectively) in a 4000-fold molar excess. Lanes 5: free probe, purified WRKY70, and unlabelled mutations S1mut1 or S2mut1, respectively in a 4000-fold molar excess. The sequences of S1, S1mut1, S2, and S2mut1 are shown. Unchanged nucleotides in S1mut1 and S2mut1 are indicated by a dash (−). A P designates the position of the free probe and a specific DNA-protein complex is marked with an asterisk (*). The gel shifts were done three times and one example is shown. The original unprocessed figures are shown in Fig. S3 and Fig. S4

To show direct binding of WRK70 to the WT-boxes CGACTTTT in S1 and S2, EMSAs were done. For this, WRK70 was expressed in *E. coli* and purified as described before [23]. Figure 11 shows a shifted complex in the presence of WRKY70 in case of sequence S2 but not S1 (lanes 3). In case of S2 this complex can be abolished or strongly reduced with an excess of unlabelled S2 (Fig. 11, lane 4). The sequence in which the WT-box is mutated (S2mut1) is no longer able to abolish the shift (Fig. 11, lane 5). In summary, WRKY 70 positively regulates MAMP-responsivity of the synthetic promoters S1 and S2 harbouring the WT-boxes CGACTTTT but direct in vitro binding was only detected to the WT-box in S2.

## Discussion

The genomic distribution analysis of the 16 different WT-boxes NGACTTTN shows a highly significant increase in the frequency of the WT-boxes AGACTTTT, CGACTTTT, GGACTTTT, TGACTTTA, and TGACTTTT in the 500 bp upstream region of the genes (Fig. 1 and Table 1). Many of them have previously been shown to be associated with MAMP-regulated gene expression [23, 35, 36]. These sequences are also part of bioinformatically predicted regulatory sequence motifs [9, 37, 38]. The analysis of the *ATEP3* minimal promoter supports the functionality of the WT-box CGACTTTT as an essential MAMP-responsive regulatory sequence (Fig. 7). When this WT-box was used in a yeast one-hybrid assay, WRKY70 was identified as a binding TF [23]. However, when using the MAMP-responsive WT-boxes GGACTTTT and GGACTTTG from a regulatory motif in the promoter of the *DJE1* gene, binding transcription factors could not be identified by yeast one-hybrid assays [36]. This led to a preliminary definition of Type I and Type II WT-boxes [39]. Later, the WT-boxes GGACTTTT and GGACTTTG from the promoter of the *DJE1* gene were shown to be bound by the WRKY DNA binding domain of WRKY50 [27]. WRKY50 was previously shown to interact with the regulatory sequence GGACTTTTC from the *PR1* promoter which harbours the WT-box GGACTTTT [26], leading to the analysis of WRKY50 binding to the WT-boxes GGACTTTT and GGACTTTG [27]. In these studies, the full size WRKY50 protein was not able to bind to its regulatory sequence in electrophoretic mobility shift assays but only the WRKY50 binding domain [26]. This was the reason why WRKY50BD was also used here (Fig. 9) for in vitro binding studies. This may also explain why WRKY50 was not selected in yeast one-hybrid screenings [36]. Previously, WRKY50BD was shown to bind to the WT-boxes GGACTTTT, GGACTTTG, GGACTTTC, and TGACTTTT [27]. Here, binding of WRKY50BD to CGACTTTT is also shown (Fig. 10). It remains puzzling why the full size WRKY50 protein can be used as an effector protein in transient expression studies in plant cells but not in in vitro binding assays. This may be due to differences in protein folding, modification, or due to cooperative binding by interacting with other TFs in vivo. Interestingly, WT-boxes have been identified as part of *cis*-regulatory modules harbouring for example a second WT-box, a bZIP binding site, a W-box or a GCC-box [26, 36, 39]. Furthermore, all WT-box containing *cis*-sequences studied for their interaction with transcription factors in parsley protoplasts were cloned as tetramers in their reporter plasmids [9, 19, 34]. This tetramerization leads to an increase in MAMP-responsive reporter gene expression compared to the monomer and dimer [36].

**Fig. 12:**
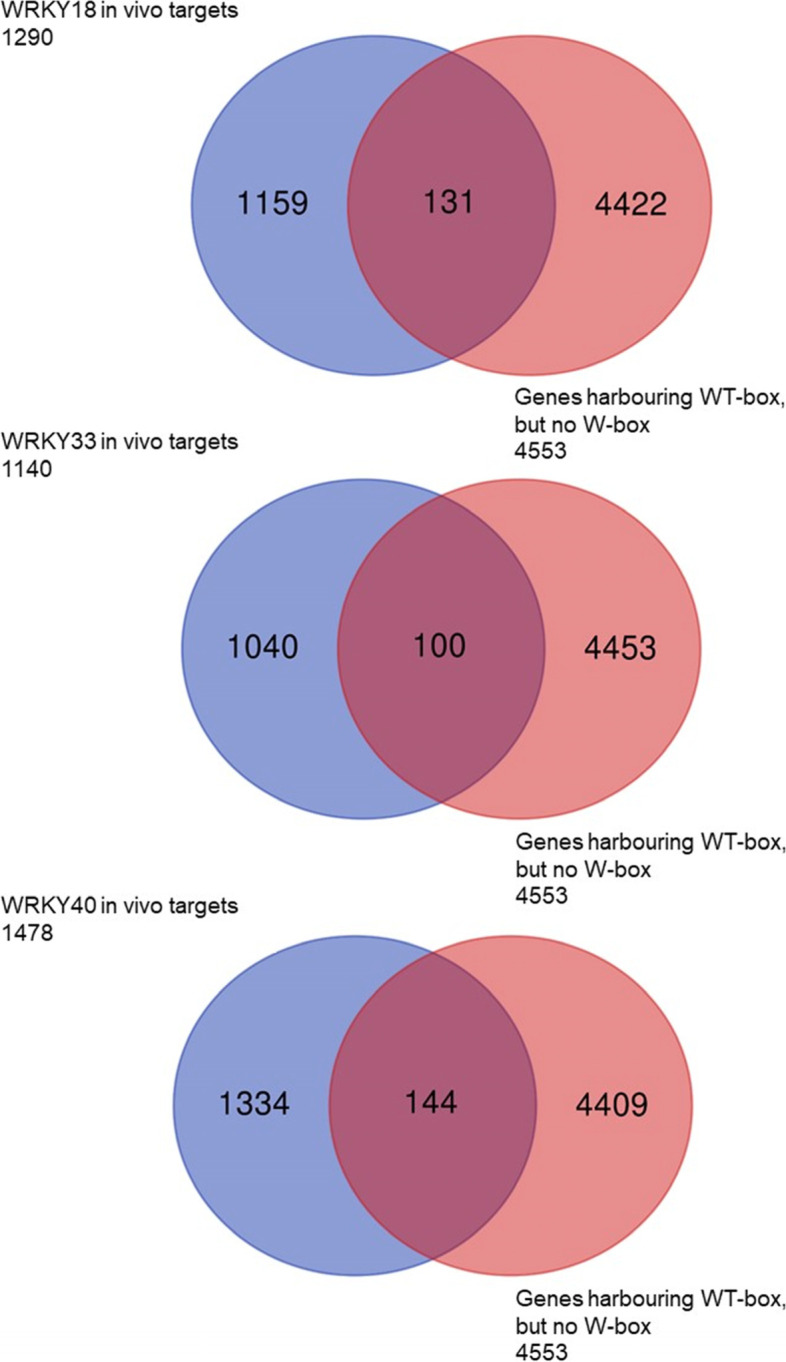
WRKY18, WRKY33, and WRKY40 target genes harbouring no W-box TTGAC/T but a WT-box NGACTTTN within their 500 bp upstream region

WRKY50 has been shown to exhibit a larger variability in its binding site specificity than other WRKY factors [40, 41]. The wide range of WRKY50 binding sites is further supported by the study here and earlier work [27]. Interestingly, many of the WRKY50 and WRKY70 binding sites deviate from the classic W-box TTGACT/C and the W-box is not part of many of these binding sites [23–27]. Therefore, it may be conceivable to propose WT-boxes not being part of W-boxes to be a missing link for WRKY regulated genes. For example, evidence for WRKY binding sites different from W-boxes comes from chromatin immune precipitation experiments using WRKY18, WRKY33, and WRKY40. For these WRKY factors in vivo binding fragments were amplified that do not harbour W-boxes [5]. To check how many 500 bp upstream regions are void of W-boxes, but contain a WT-box the PatMatch tool at the TAIR web site was used and led to the identification of 4553 genes [42]. Comparing those to the target genes of WRKY18 (1290), WRKY33 (1140), and WRKY40 (1478) determined by chromatin immunoprecipitation after 2 h flagellin treatment [5], many genes with WT-boxes but no W-boxes in their 5′ region were identified. Figure 12 shows a Venn diagram identifying 131 (WRKY18), 100 (WRKY33) and 144 (WRKY40) in vivo WRKY target genes harbouring a WT-box but no W-box within their 500 bp upstream region. Based on these findings, WT-boxes, not being part of the classic W-box, may enrich the repertoire of WRKY binding sites.

It may be interesting to ask, why WT-boxes without W-boxes have not been identified as WRKY binding sites before. The W-box was originally identified as the sequence TTGACC in the W1- and W2-boxes of the parsley *PR1–1* and *PR1–2* promoters [22]. When the sequence TTGACC in these W-boxes is mutated, their function is abolished. The W3-box contains the core sequence TGAC required for promoter function [22, 43]. With these W-box sequences, the first WRKY factors were isolated from a parsley expression library [22]. Later, the W-box TTGACC/T was established as the minimal consensus sequence required for specific DNA binding [1]. The binding of specific WRKY factors with the W-box depends on the presence of nucleotides adjacent to this sequence [44]. To identify a wider range of WRKY binding sequences random binding site selection experiments in which WRKY factors were used to select randomly generated oligonucleotides are useful. The first WRKY factor from *Arabidopsis thaliana*, ZAP1 (WRKY1), which was employed for in vitro random binding site selection, identified the consensus binding sequence CGTTGACCGAG which is present in most of the bound sequences [45]. The sequence with the highest binding affinity was determined to be TTGACCGACTTGACTTTTA, harbouring two W-boxes TTGACT/C of which one happens to be part of the WT-box TTGACTTTT. Interestingly, the sequence with the lowest binding affinity to WRKY1 does not harbour a W-box but the WT-box AGACTTTT [45]. Therefore, WT-boxes not being part of the classic W-box may be weaker binding sites for WRKY factors and may require additional *cis*-sequences for their functionality. As discussed above, WT-boxes are often part of combinatorial elements. Furthermore, the stretch of four T’s may be a factor for WRKY binding site recognition. Recently, A/T-rich modules flanking the MYC-binding motif, were shown to be essential for TF recognition [46]. Because all WT-boxes are characterized by a stretch of T’s this may contribute to the binding of WRKY factors to WT-boxes not being part of a classic W-box.

## Conclusions

Many WT-boxes, particularly those not directly linked to a W-box, are significantly enriched in promoter regions, are MAMP-responsive, and are novel WRKY binding sites. Based on previous chromatin immunoprecipitation experiments using WRKY factors, a large number of promoters of these target genes do not harbour a W-box but a WT-box. Therefore, the WT-box is a missing link for the identification of WRKY target genes, especially when target genes do not harbour W-boxes.

## Methods

### Genomic distribution analysis of WT-boxes and the identification of a WT-box rich upstream region

To determine the genomic distribution of all 16 WT-boxes, the PatMatch online tool at TAIR was used: https://www.arabidopsis.org/cgi-bin/patmatch/nph-patmatch.pl [33, 42]. All 16 WT-boxes NGACTTTN were submitted individually and searched against the “TAIR10 Whole Genome Sequence Database” to determine the absolute number of WT-boxes in the whole genome. The number and position of the 16 WT-boxes NGACTTTN in the whole genome determined with the “TAIR10 Whole Genome Sequence Database” is shown in Table S1. To determine the occurrence of WT-boxes 500 bp upstream, the same searches were performed with the “Araport11 Loci Upstream Seq – 500 bp (DNA)” sequence database [47]. From all hits found in the “Araport11 Loci Upstream Seq – 500 bp (DNA)” sequence database, genes located within the chloroplast and mitochondrial genomes were subtracted. The number and position of the 16 WT-boxes NGACTTTN 500 bp upstream of all genes determined with the “Araport11 Loci Upstream Seq – 500 bp (DNA)” sequence database is shown in Table S2. Table 1 shows the numbers of hits obtained with these two searches. To determine the frequencies of WT-boxes, the expected frequencies were determined under consideration of the A/T and G/C content within the 500 bp upstream region of all genes. For example, the raw data from TAIR contains 33,323 genes, times 500, yields the nucleotides for which the GC content was determined. This was 32.62%. Therefore, G or C occur in a frequency of 0.1631 each and A or T occur in a frequency of 0.3369 each. This means that the expected frequency of the WT-box AGACTTTA is 0.3369^6^ × 0.1631^2^ = 3.88968E-05. This was done for all 16 WT-boxes (Table 1). The observed frequencies of all WT-boxes either genome wide or within the 500 bp upstream regions was determined by dividing the observed numbers (Table 1) to the region analysed for their occurrence. To determine the statistical significance of the obtained frequencies the function BIONOM.DIST in excel was employed using a confidence interval of 95%. This yields a *p* value that determines if a certain frequency can occur randomly or is significantly higher than expected. A *p*-value < 0.5 shows a highly significant deviation from the expected frequency.

To identify a gene with many WT-boxes, PatMatch was employed for identification of the WT-box core sequence GACTTT within a 1000 bp upstream region using the “Araport11 Loci Upstream Seq – 1000 bp (DNA)” sequence database. Only two genes were identified to harbour the maximum number of seven WT-box core sequences in this region. Of these two, *ATEP3* (At3g54420), a class IV chitinase was chosen for further WT-box analysis because it is upregulated by a large of number of pathogenic stimuli [48]. The sequence of the upstream region was extracted from TAIR and the position of all seven WT-boxes relative to the transcription start site were determined. Similarly, W-box free upstream regions harbouring a WT-box were identified with PatMatch. First all nuclear genes harbouring a W-box TTGACC or TTGACT within their 500 bp upstream region were identified. Then all nuclear genes harbouring the WT-box core sequence GACTTT within their 500 bp upstream region were identified. Both lists of genes were compared to generate a list of 4553 nuclear genes harbouring a WT-box NGACTTTN but not a W-box TTGACY within their 500 bp upstream region. A list of 4553 nuclear genes harbouring a WT-box NGACTTTN but not a W-box TTGACY within their 500 bp upstream region is shown in Table S3. The lists of target genes for WRKY18 (1290), WRKY33 (1140), and WRKY40 (1478) determined by chromatin immunoprecipitation after 2 h flagellin treatment was obtained from an earlier paper [5]. These gene lists were used to determine WRKY18, WRKY33, and WRKY40 target genes not harbouring a W-box but a WT-box in their 500 bp upstream region. Venn diagrams were generated at https://bioinformatics.psb.ugent.be/webtools/Venn/.

### Plasmids

All *cis*-sequences harbouring WT-boxes and their mutations were synthesized as 34 bp long monomers or dimers with partial SpeI and XbaI sites by Life Technologies (Darmstadt, Germany). All sequences without the linker sequences are shown in the figures. The single stranded sequences were annealed and ligated into the SpeI/XbaI sites of pBT10GUS-d35SLUC [9]. Tetramerization was done as described [20]. Recombinant reporter plasmids harbouring the tetramer of sequence S20 and S22 were described previously [9]. Reporter plasmids harbouring upstream fragments from *ATEP3* were constructed by cloning commercially synthesized DNA fragments (BioCat GmbH, Heidelberg, Germany) into pBT10GUS-d35SLUC by first removing the TATA-box of the *uid*A minimal promoter from the plasmid as described before [36]. The construction of effector plasmids expressing WRKY50 (WRKY50-pORE) and WRKY70 (WRKY70-pORE), used in transient reporter gene expression assays, have been described [23, 27].

For gel shift assays WRKY50BD cloned in pQE-30 and WRKY70 cloned in pQE-32 were expressed in *E. coli* BL21 [23, 27].

Plasmid DNA for protoplast transformation was isolated as described by the manufacturer with the ‘NucleoBond^R^ xtra midi EF’ or ‘NucleoBond^R^ xtra maxi kit’ (Macherey-Nagel, Düren, Germany). For all recombinant DNA work, standard protocols were employed [49]. All cloning products were sequenced by Microsynth Seqlab (Göttingen, Deutschland). DNA sequences were processed and analysed using the ‘CLC Main Workbench’ software (CLC Bio, Aarhus, Denmark).

### Transient reporter gene expression in parsley protoplasts

A cell suspension culture of parsley (*Petroselinum crispum*) was used for transient reporter gene expression analysis. The cultivated cells were used for protoplast preparations and freshly prepared for each transformation experiment according to a published protocol [20]. For co-transformation experiments a TF-expressing effector construct based on pORE-O2-d35S-pA and reporter gene constructs harbouring the *cis*-regulatory sequences cloned as tetramers upstream of the minimal promoter of the *uidA* gene in pBT10GUS-d35SLUC (pBT10) were performed as described [23, 27]. MAMP-responsive reporter gene assays were done using recombinant pBT10GUS-d35SLUC reporter gene constructs with or without co-transformation of a TF-expressing effector plasmid into freshly prepared parsley protoplasts. Subsequently the transformed protoplasts were treated with or without the MAMP Pep25. As a positive control, the D-element cloned as a tetramer into pBT10GUS-d35SLUC was used [9]. Quantification and normalization of reporter gene expression was done according to a published protocol [20]. All transformations were done at least three times independently with two technical replicates each. The exact number of experiments from which mean values and standard deviations were derived are given in the figure legends. Statistical differences between experiments were determined with a t-test in microsoft excel. An asterisk in the figures denotes a significance threshold of ≤0.05 between two experimental conditions. Statistically significant differences are indicated with one (*p* < 0.05), two (*p* < 0.01), or three (*p* < 0.001) asterisks.

### Electrophoretic mobility shift assays

Expression and purification of recombinant WRKY50BD and WRKY70 from *E. coli* and binding reactions to biotin-labelled probes and gel electrophoresis were done according to earlier protocols [23, 27]. Biotin-labelling of the probes was done using the ‘Biotin 3’ End DNA Labelling Kit’ (Thermo Fisher Scientific). Probe and competitor sequences are shown in the corresponding figures. Blotting, crosslinking of the probe to the membrane, and probe detection was done according to the manual of the ‘LightShift Chemiluminescent EMSA Kit’ with a ChemiDoc™Touch Imaging System (BioRad). For each experiment the results were confirmed independently.

## Supplementary Information

**Additional file 1: Table S1.** The number and position of the 16 WT-boxes NGACTTTN in the whole genome determined with the “TAIR10 Whole Genome Sequence Database”.

**Additional file 2: Table S2.** The number and position of the 16 WT-boxes NGACTTTN 500 bp upstream of all genes determined with the “Araport11 Loci Upstream Seq – 500 bp (DNA)” sequence database.

**Additional file 3: Table S3.** A list of 4553 nuclear genes harbouring a WT-box NGACTTTN but not a W-box TTGACY within their 500 bp upstream region.

**Additional file 4: Fig. S1.** Binding of WRKY50BD to the sequence S1. Original unprocessed figure with multiple exposures.

**Additional file 5: Fig. S2.** Binding of WRKY50BD to the sequence S2. Original unprocessed figure with multiple exposures.

**Additional file 6: Fig. S3.** Binding of WRKY70 to the sequence S1. Original unprocessed figure with multiple exposures.

**Additional file 7: Fig. S4.** Binding of WRKY70 to the sequence S2. Original unprocessed figure with multiple exposures.

## Data Availability

The datasets generated in the current study are included in this article. The author responsible for distribution of materials integral to the findings presented in this article is Reinhard Hehl.
