# Supplementary figures and images for: Genomic distribution and context dependent functionality of novel WRKY transcription factor binding sites
Source: BMC Genomics. 2022 Sep 27;23:673. doi: 10.1186/s12864-022-08877-y (PMC9513909; doi:10.1186/s12864-022-08877-y)

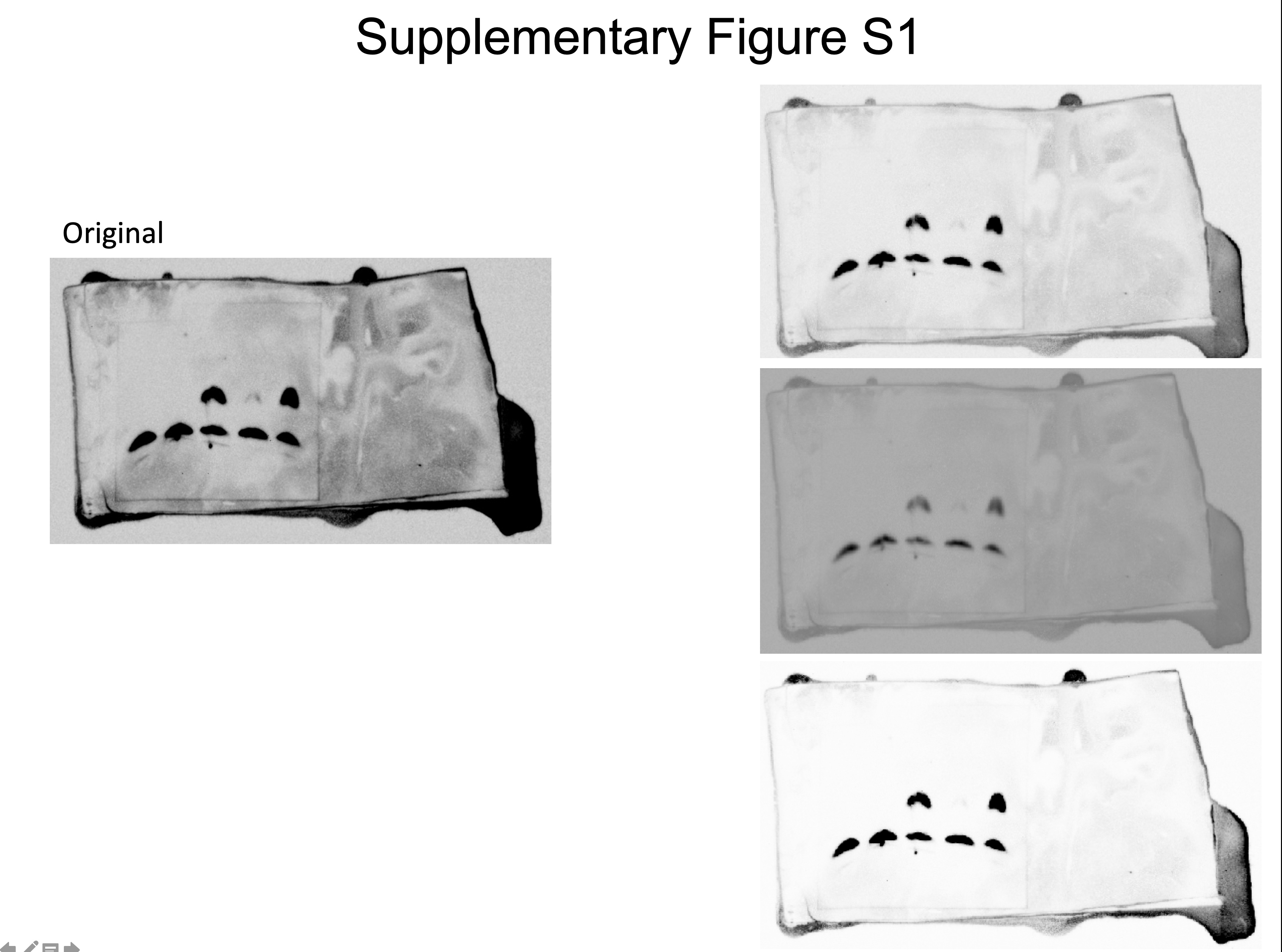

Supplement: Supplementary file 4 — Additional file 4: Fig. S1. Binding of WRKY50BD to the sequence S1. Original unprocessed figure with multiple exposures. [file 12864_2022_8877_MOESM4_ESM.jpg]

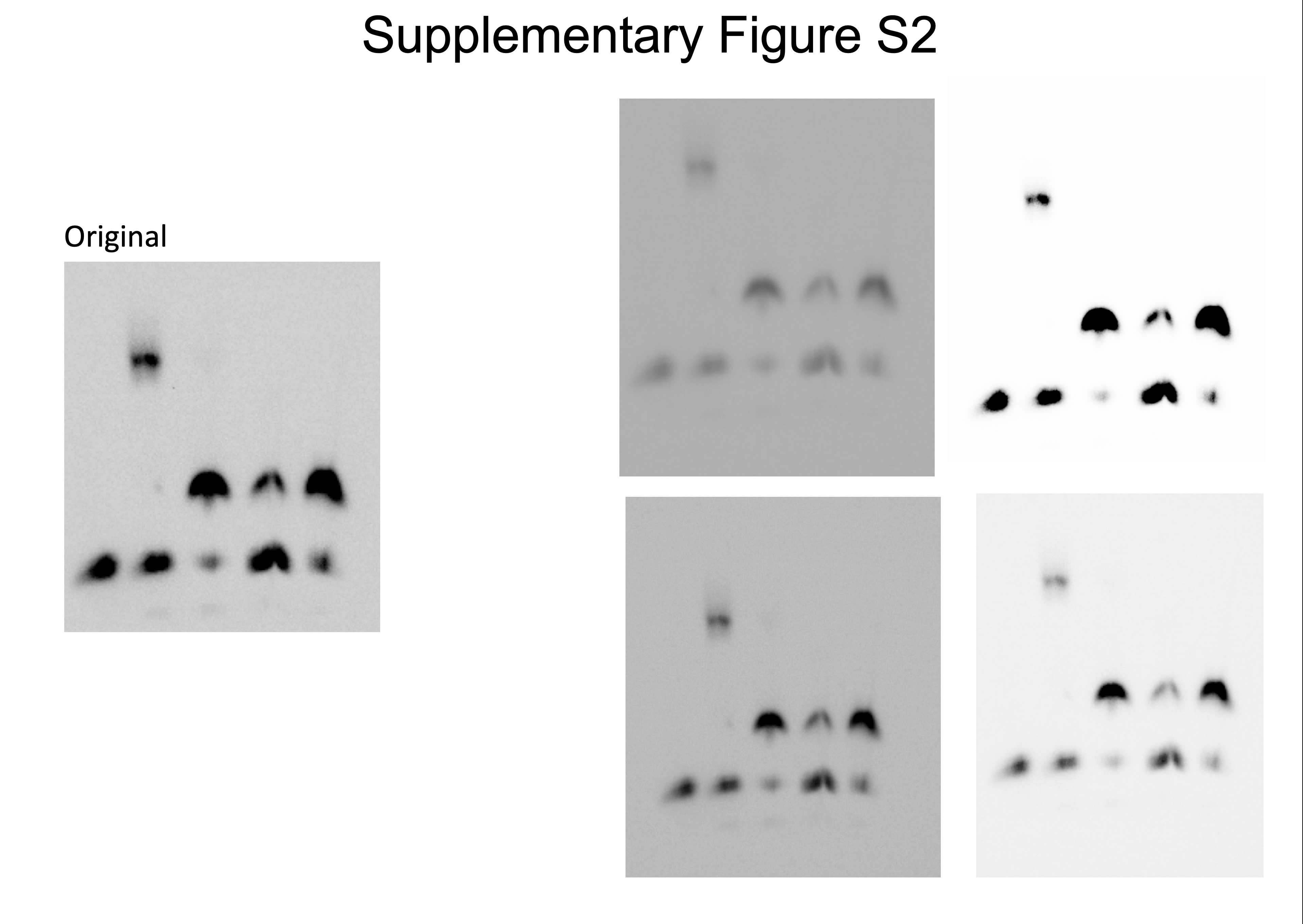

Supplement: Supplementary file 5 — Additional file 5: Fig. S2. Binding of WRKY50BD to the sequence S2. Original unprocessed figure with multiple exposures. [file 12864_2022_8877_MOESM5_ESM.jpg]

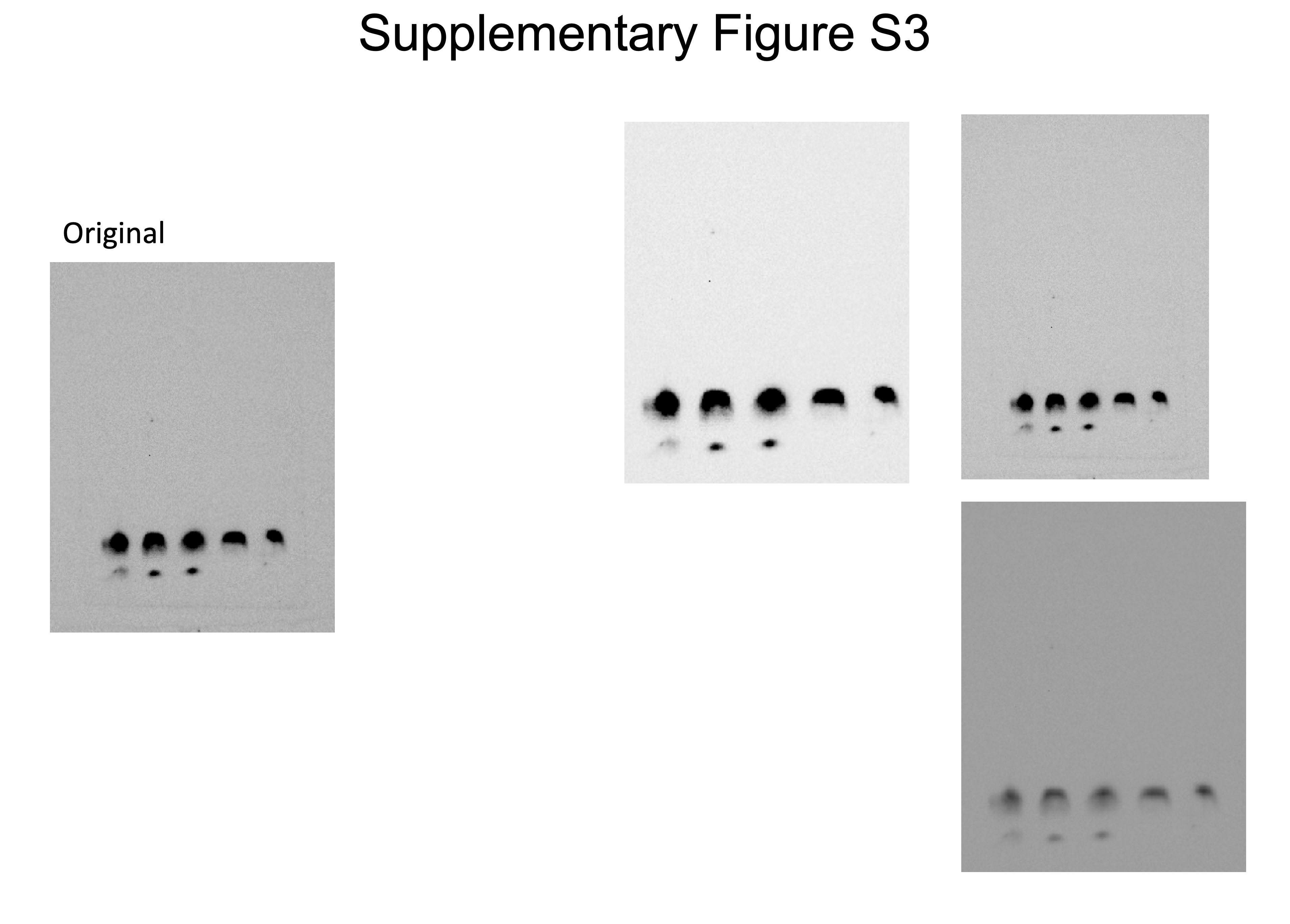

Supplement: Supplementary file 6 — Additional file 6: Fig. S3. Binding of WRKY70 to the sequence S1. Original unprocessed figure with multiple exposures. [file 12864_2022_8877_MOESM6_ESM.jpg]

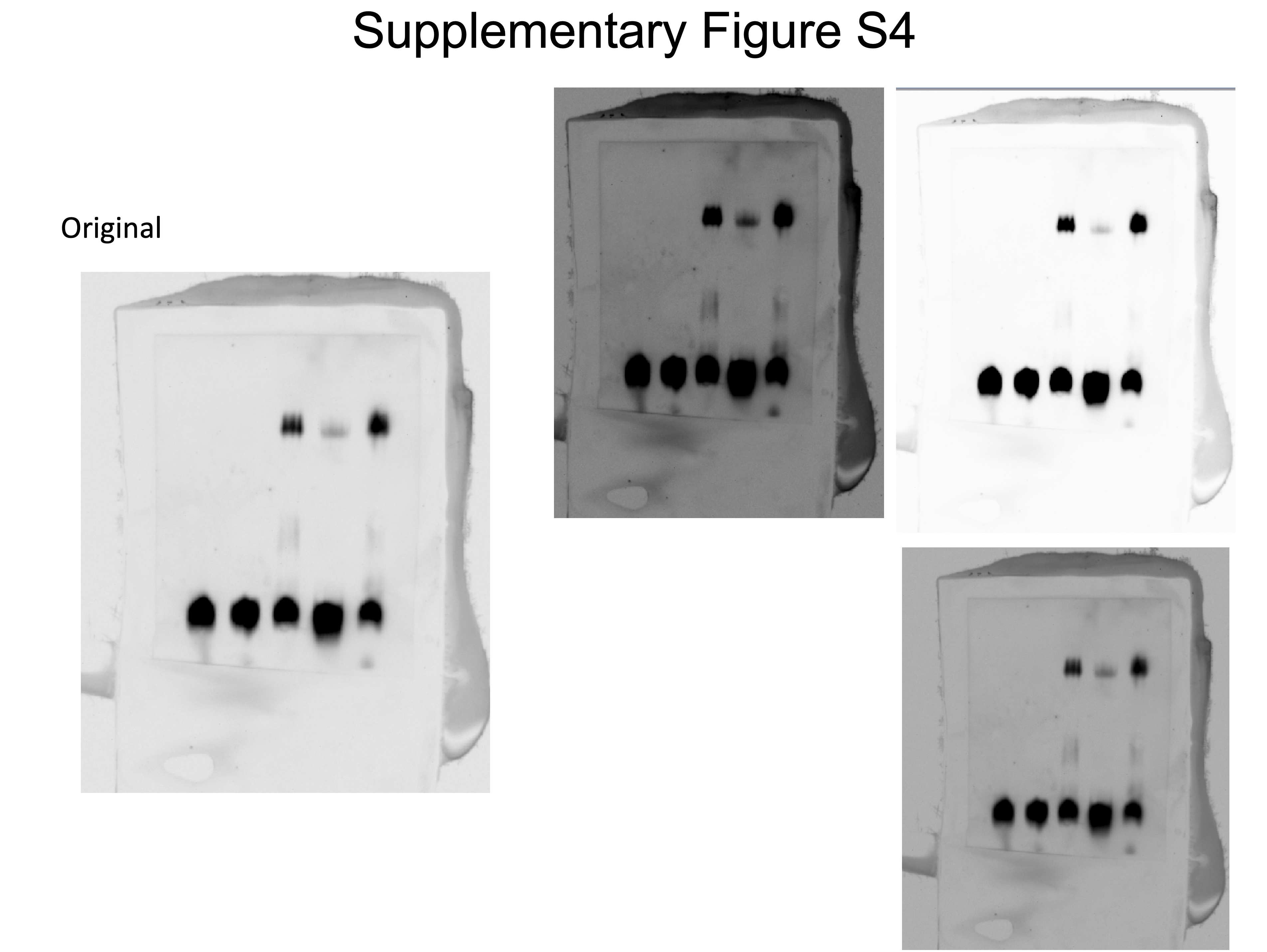

Supplement: Supplementary file 7 — Additional file 7: Fig. S4. Binding of WRKY70 to the sequence S2. Original unprocessed figure with multiple exposures. [file 12864_2022_8877_MOESM7_ESM.jpg]
